# Early Career Perspectives from Large Pharma, Software, and Start-up Companies

**DOI:** 10.1021/acs.jcim.1c01416

**Published:** 2022-05-19

**Authors:** Fiorella Ruggiu, Caitlin Bannan, Andrea Bootsma

**Affiliations:** † insitro Inc., 279 East Grand Avenue, South San Francisco, California 94080, United States; ‡ OpenEye Scientific Software Inc. 9 Bisbee Court, Suite D, Santa Fe, New Mexico 87508, United States; § Pfizer Medicine Design, 610 Main Street, Cambridge, Massachusetts 02139, United States

## Abstract

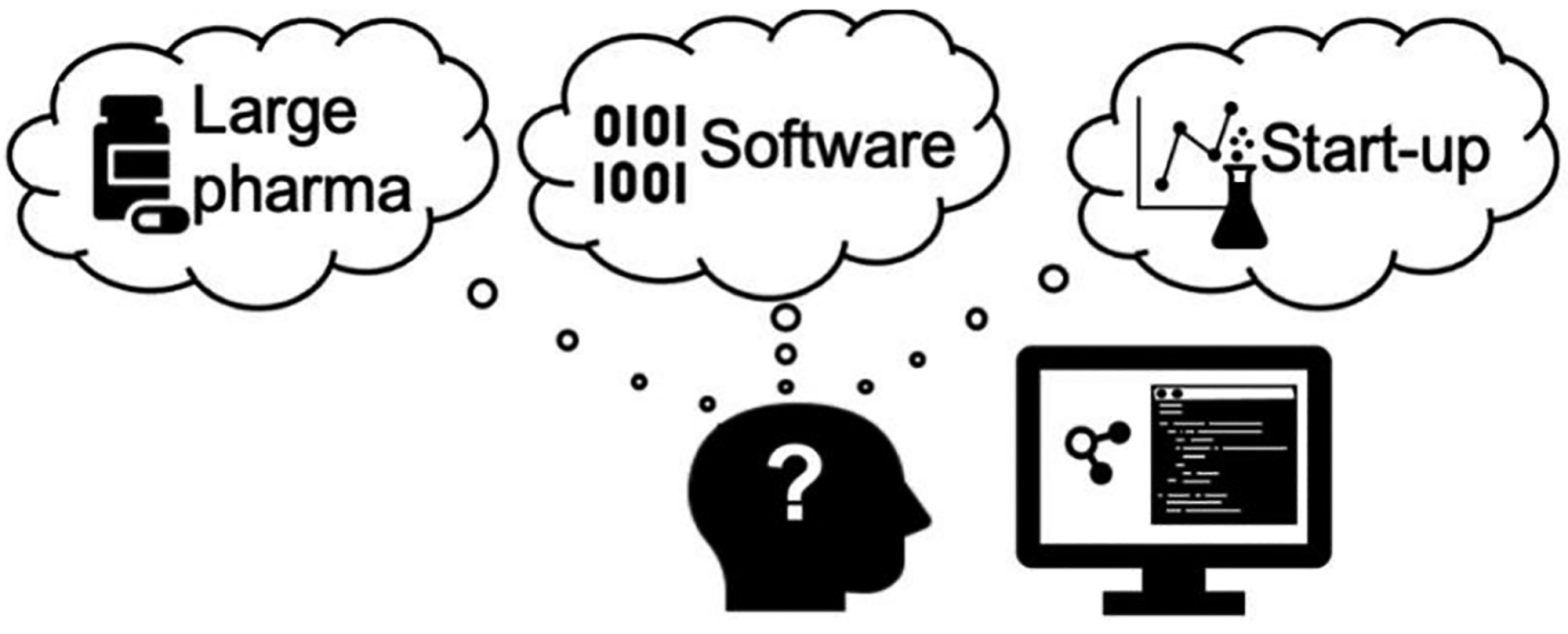

We are often approached by PhD students and postdocs who wonder: What are the differences between jobs for computational chemists across different industries? This Perspective aims to answer this question by comparing our personal experiences as early career scientists at a large pharmaceutical company (large pharma), a software vendor (software), and a biotech start-up (start-up) in the format of a written Q&A panel discussion. To begin, we introduce ourselves by answering questions about our backgrounds and current positions, including comparisons of our responsibilities and the culture of the companies where we work. In the next section, we focus on the beginning of our careers, discussing the skills we needed for our first industry positions and what we learned early on. Finally, we address questions about the future of our careers including potential growth, security, and what we wished we had known earlier. We conclude by comparing and contrasting our industries, including how the size and purpose of these companies have affected our experiences.

## Introduction

Computational chemistry has evolved into an important part of the pharmaceutical industry and is expected to continue to grow, making it an area of interest to many early career scientists.^1−3^ Those scientists tend to look for resources about the different careers for computational chemists in industry. One such resource is a special issue from the *Journal of Computer-Aided Molecular Design* titled “Computer-aided drug design (CADD) strategies in pharmaceutical research”.^4^ Many articles in that issue highlight the role, responsibilities, and importance of computational chemists at pharmaceutical companies. Beyond the pharmaceutical industry, there exists a multitude of other opportunities for computational chemists in different industries, for example, in astrochemistry as Herma Cuppen discussed in an interview about her career^5^ or in climate change as described by John Keith.^6^

Individual interviews provide an insight into the motivations and daily life of computational chemists with varied backgrounds and career paths.^7,8^ In particular, the European Federation for Medicinal Chemistry and Chemical Biology^8^ has several interviews with various computational chemists on their Web site (Computational chemists interviewed on the European Federation for Medicinal Chemistry and Chemical Biology Web site include: Zaid (M. Jaber) Al-Obaidi, Xavier Lucas, György Ferenczy, Victor Sebastian Perez, Giovanni Bottegoni, Gisbert Schneider, Ferran Sanz, Filipa Ramilo Gomes, Ana Sofia Newton, Davide Benedetto Tiz, Ana Amic, and Antti Poso.). In addition, there have been a few perspectives on the transition from academia to industry in recent years,^9,10^ including one from Kendall Powell, which focuses on practical advice on the industry interview process.^11^ While all of these resources are helpful for a person transitioning into industry, they tend to focus on an individual company or person, making it difficult to directly compare the different sectors.

We wrote this Perspective to provide a resource to compare the different positions of computational chemists in the pharmaceutical industry. A common question we receive from PhD students and postdocs is what are the differences between jobs for computational chemists across different industries? We set out to answer the questions we wished we’d had answers to during our own job searches. This Perspective is limited to our personal experiences, focusing on the pharmaceutical industry in the United States. However, we hope that our experience can still highlight larger trends, demonstrate several available paths, and help you decide what factors are important for your job search.

This Perspective aims to give a comparison of our personal experiences as early career scientists in a large pharmaceutical company (Large Pharma), a software vendor (Software), and a biotech start-up (Start-up) in the format of a written Q&A panel discussion, which allows us to share specific details and personal perspectives. However, it is important to keep in mind that, while some answers can be generalized to the industry experience, others may be more reflective of specific locations or companies, and we try to provide sufficient context for the reader to determine which aspects of what we share are likely to be relevant to their specific situation. We share background on our roles and companies, the experience of transitioning to industry and starting in a new role, and our early experiences with career development and progression. Our hope is that this perspective will give PhD students and postdocs insight into what a career in each of these sectors can look like.

## Panel Discussion

### Introduction and Background

#### What Is Your Background?

##### Andrea (Large Pharma)

“My PhD was focused on using density functional theory and other quantum mechanical methods to develop molecular descriptors and quantify trends in intermolecular interactions. I joined Pfizer’s Medicine Design group immediately after graduating from the University of Georgia in May of 2019, starting out as a Senior Scientist in computational chemistry supporting medicinal chemistry projects in Inflammation & Immunology, and this is the perspective from which I will be answering these questions. There are other areas for computational chemists within Pfizer, such as those who work more on the method development side or who are focused on process chemistry or the prediction of properties of bulk materials, and people who work in those areas may have a different experience. Pfizer itself is a large company, founded over one hundred years ago and employing over 70 thousand people worldwide. As a company, we are focused on delivering ‘breakthroughs that change patients lives’ across several disease areas and therapeutic modalities. There are multiple Pfizer locations around the country, and I am based at the site in Cambridge, MA, which has about 1000 employees. This also influences the perspective I am able to provide.”

##### Caitlin (Software)

“I received my PhD from the University of California, Irvine, where I worked with the Open Force Field Initiative to automate chemical perception during small molecule force field parametrization. During that time, I also did an internship with OpenEye Scientific Software Inc. (OpenEye) where I built machine learning (ML) models for small molecule p*K*_a_ predictions. I joined OpenEye as a Scientific Software Developer immediately after completing my PhD from the University of California, Irvine, in August 2019. At OpenEye, the scientific research teams are divided on the basis of discipline (i.e., cheminformatics or biomolecular modeling). I am a part of the physics group that develops quantum chemical work flows and researches and develops methods for small molecule crystal structure prediction (CSP). OpenEye is a relatively small company (∼100 employees currently) headquartered in Santa Fe, NM, with offices in Boston, Tokyo, and Cologne. It was founded in 1997 to develop software for large-scale molecular modeling primarily applied to drug discovery. In recent years, OpenEye has expanded significantly with the introduction of Orion, a cloud computing platform for drug design. Most of my work focuses on using or developing for Orion. There are many other types of computational chemistry positions in software companies, such as application scientists who interface with customers. My experience as a developer will strongly influence my perspective and answers to the questions below.”

##### Fio (Start-up)

“I did my PhD on fragment descriptors and used them to build ML models for various drug discovery relevant properties at the laboratory of cheminformatics in Strasbourg. I followed with a postdoc at Novartis Institutes for BioMedical Research (NIBR) in the Computer-Aided Drug Design team within the chemistry department and focused on matched molecular pairs analysis in regard to the effect of intramolecular hydrogen bonds on permeability, ensemble of conformers with NMR data, and Gram-negative entry of a molecule considered ‘too large’ for permeation into the pathogen. I then joined DiCE Molecules (DiCE, now DICE Therapeutics) in December 2018, a small established start-up in DNA-encoded libraries (DEL). There, I worked as a cheminformatics scientist where I worked on DEL data analysis and hit picking, enumeration of libraries, design of building blocks, and more. The company was established in 2013 and had about 30 employees while I was there. There were 2 active medicinal chemistry programs, 2 partnerships, and major work in running DEL selections against many targets. I currently work in the drug discovery department at insitro, founded in 2018; I joined in February 2020 as a cheminformatics and computational chemistry scientist. The company had about 60 employees then and has since grown to over 100. It is a fast paced start-up focused on bringing machine learning to bear on the overall drug discovery pipeline, in particular by identifying more relevant targets to diseases, with a focus on liver and neuro diseases, and performing screens leveraging pluripotent stem cells. My answers will mainly be reflective of my experience at insitro.”

#### How Was Your Job Search after Graduating?

##### Caitlin (Software)

“It is important to note here that the job search process is going to be different for every person, regardless of industry. I had worked outside academia before graduate school, and I always knew I wanted to pursue a career in industry. Originally, I planned to apply for industry postdoc positions, which seemed like a good way to transition from academia to industry. During the final year of my PhD, contacts in my network indicated that I could likely find a full time position in industry without doing a postdoc. One common reason they cited was that my graduate research was focused on problems directly applicable to the pharmaceutical industry. Due to interest from people in my network, I began applying to positions about nine months from my anticipated defense. Most of the positions I applied to were thanks to people in my network. In some cases, I had collaborated directly with the company, as with OpenEye, but for some positions, I only had contact from networking at conferences. One thing that surprised me in this process was the interest from industry when I was still many months from graduation. Due to preparing so far in advance, the start date was an important part of my negotiation. I applied to both postdoc and full time positions in both software and pharmaceutical companies. The application and interview process helped in my understanding of the difference in these industries. Like any industry, the demand for new employees will fluctuate over time. At the time of my job search, there was high demand for computational chemists and I was able to leverage my network to help me find a position that was right for me.”

##### Andrea (Large Pharma)

“I started applying for jobs about six months before I was planning to graduate. However, before I started applying, I spent about two months consistently checking the job postings on sites like Linkedin and Indeed, reading job descriptions and preferred qualifications, and getting a sense of the state of the job market at that time. I had gotten mixed feedback on whether a postdoc was required before starting in industry and so, like Caitlin, I applied simultaneously to industrial postdoc programs and full time positions and ultimately did obtain a full time position without a postdoc. Unlike Caitlin, I did not have a lot of contacts in industry in my network, and so I relied heavily on online job sites for which to find openings to apply.”

##### Fio (Start-up)

“Unlike Caitlin and Andrea, I finished my PhD 5 years earlier and there seemed to be very few positions for computational chemistry in research in Europe. I initially wanted to stay in France. I wasn’t sure what I wanted to do for my future career and needed time to figure it out, which slowed down my search considerably. Eventually, I became certain that I wanted to work in pharmaceutical research and what would fit me best was an industrial setting. This was a surprise as I had started my PhD thinking I would continue in academia. I came to that conclusion after having an extensive conversation with a very senior cheminformatician working in industry. I believe it is very important to talk to professionals and ask questions to figure out one’s next steps throughout our careers. I focused my search for positions corresponding to my criteria and was told by several people that a postdoc was necessary. The small number of positions in France and even in Europe made the search difficult. I eventually heard of the postdoc opportunity at Novartis, which was an extremely appealing fit for me and decided to take my chances. I was offered the position and took the jump to move to the US. Making that choice wasn’t easy, but I have never regretted it. Looking back, I had not been ready to make such a move until I had that offer. It could have been good to explore the global market earlier in my search. I also think that the market has changed and it’s easier now to get a position in industry without a postdoc, and also, more remote work opportunities are available.”

#### What Are Your Responsibilities and What Does Your Average Day Look Like?

##### Andrea (Large Pharma)

“The big picture description of my work is to impact ongoing drug discovery projects. On a more practical level, this means using whatever tools are appropriate to help generate new ideas and prioritize others’ ideas on the basis of available information and acting as an interface between structural biology and medicinal chemistry. Altogether, project work is the focus of about 85% of my time. I also participate in efforts to bring in and evaluate new computational tools, which is how I spend the remaining 15% of my time. My day to day consists of running analysis for a project including docking, scoring, torsional analysis, molecular dynamics, or some combination as needed and then processing these results and preparing communication about them with the project team. About 25% of my time is meetings with project teams: either to get information about project status or to work through design ideas and present results.”

##### Caitlin (Software)

“My work is split between two main responsibilities, software development and research, both with the goal of bringing physics based solutions to a wide variety of problems our customers face. I manage our quantum chemical based workflows on Orion. This is primarily a software development task that includes planning new features, coding, testing, and writing documentation for that product (40%). My research is currently focused on how to expand and improve our protocols for small molecule CSP (60%). This has felt like a more pragmatic version of research in graduate school. It includes comparing our predicted crystal structures with experimental structures and using those results to drive improvements to our methods in both accuracy and efficiency.

About 20% of my time is spent in meetings, which is due to the collaborative nature of our work. On the research side, we tend to share results frequently and make many decisions as a team. With software development, we work as a team to test both our workflows and the user interaction with our products. Outside of meetings, we have a lot of discretion in how to distribute our time day to day. I enjoy having a bit of diversity in my day to day, so I usually try to switch between tasks when I’m able. To make sure I’m staying on top of deadlines, I usually try to establish a couple goals for the week, both in my software responsibilities (i.e., pick a couple bugs to fix) and on the research side (choose one question or experiment to address). Then, I’ll alternate between those when I need a change of pace. An upcoming deadline will also drive those priorities. For example, when we are near an Orion release, most of my time will be spent testing or writing documentation for that release.”

##### Fio (Start-up)

“I contribute to various projects that range from chemical screens to machine learning building with cheminformatics workflows or data analysis, for example, selecting compounds for a particular screen or enumerating a DEL. The work is a mix of project and general meetings (35%), planning and leading projects I’m responsible for (15%), coding and building infrastructure (25%), planning and doing research (20%), and corresponding with partners and vendors (5%). The exact mix of time spent on these tasks each day or week varies depending on the current, most urgent priorities. Responsibilities can shift rapidly in this environment as projects change priority or new hires are on-boarded. The pandemic did result in an increase of meetings to keep in touch.”

#### With How Many Different Groups Do You Regularly Interact?

##### Fio (Start-up)

“I am usually working with 5 different groups and am on 5 projects that range from drug discovery projects to infrastructure builds. What I focus on varies a lot over time. I interact with the ML team for building models relevant to drug discovery, our internal DEL efforts, and our phenotypic screening teams mainly to source compounds as well as with our software engineers to build infrastructure for compounds and from time to time with leadership. I also interact with vendors for compounds and software purchases as well as our partners in the collaboration with DiCE, my previous company.”

##### Andrea (Large Pharma)

“Similarly, I meet with many different groups, but differently, they are very rarely from outside Pfizer. Like Fio, I support projects, usually 2–3 drug discovery projects at one time, where I interact most closely with the design lead and 1–2 other designers. I also interact with team members from a wide variety of disciplines, including synthesis, structural biology, and pharmacokinetics, dynamics, and metabolism in meetings that vary in size as a project progresses. While project responsibilities change over time, people are typically supporting the same project for at least a year and more often longer than that. I also meet regularly with computational chemists who support other projects and the computational scientists to share lessons learned from our respective projects or work on development and implementation of internal tools.”

##### Caitlin (Software)

“I also interact with a variety of groups in and outside of our company. These meetings range in size from one on one meetings with my manager and small group meetings (∼5 people) that focus on very detailed research or software next steps to large meetings with all developers in the company to learn about big picture goals and test new versions of our software. Different from Fio or Andrea, if I am interacting with people outside our company, they will almost always be customers who use our software. These interactions include tutorials about the Orion part I work on, conversations to get feedback, and research collaborations. When in the office, I usually try to eat lunch or get coffee with coworkers from all different teams where we may or may not discuss work.”

#### How Is the Culture?

##### Caitlin (Software)

“Overall, I would describe the culture at OpenEye as friendly and opinionated. We spend a lot of time testing software and are encouraged to be critical of every new feature being introduced. Many people spend time socializing with coworkers as well. We are a medium sized company in a pretty small city. Many employees move to Santa Fe for work, so there is a lot of camaraderie amongst colleagues.”

##### Fio (Start-up)

“The cultures between NIBR, DiCE, and insitro varied a lot but all my colleagues were always welcoming and helpful. NIBR had many possibilities for exploration and many colleagues to interact with across the globe. DiCE was a small company with 30 people when I joined. There was a much bigger sense of camaraderie, and my team was very focused on a particular task. Insitro is a newer and fast growing biotech company and has a ‘techie’ flavor to it as there are many software engineers and ML engineers. There is a focus on programming and computational applications, which may be atypical for a drug discovery company. There is a standing happy hour every 2 weeks. We also have weekly all hands meetings to update everyone on what is going on. It usually includes a scientific seminar. In my opinion, a company’s structure and culture is very much in flux while growing and also as procedures become more established.”

##### Andrea (Large Pharma)

“The culture in my group is overall professional but friendly. Everyone is encouraged to speak up and share ideas. Many people have been there a long time and experienced a lot of organizational changes. They demonstrate how to weather those changes well and keep the focus on the project and the science. People’s experiential knowledge is really valued, and so, there are a lot of opportunities such as seminars or internal symposia to learn from your colleagues. Additionally, there are opportunities for fun such as monthly mixers within the medicine design group as well as site-wide events for all the groups at this office.”

#### What Do You Think Is Unique in Your Environment?

##### Fio (Start-up)

“The start-up environment tends to be fast-paced and wide-ranging. You have to take on a large variety of tasks and responsibilities, including in areas that may be totally new to you, in part because of the smaller team size. That smaller team size combined with a flatter organizational structure means that I have the opportunity to talk to our leadership more often than at large companies, such as NIBR. In the specific context of insitro, our location and ML focus means we have also adopted some culture features from the tech world and have some of those workplace perks like catered lunch.”

##### Caitlin (Software)

“Similarly, OpenEye is still a relatively small company where I frequently have contact with the leadership. In addition to the size of the company, the culture is affected by being a software company that includes many of the tech style perks, such as providing coffee and food in the office. The fact that we are not doing our own drug development and are instead developing tools for others means we have a more general approach to how we think through problems. We have to consider all the different ways a user might interact with the software and what features would help a large variety of people and the problems they face. This is a different situation than doing software development internally for a drug discovery company where you are more focused on a particular problem and have direct access to all of your users. Our culture is very community focused, both in terms of community amongst the employees and opportunities to support the local community.”

##### Andrea (Large Pharma)

“Pfizer’s structure specifically has some unique features, including distinct roles for design chemists and synthetic chemists, who combined with a computational chemist generally make up a project team. Computational chemistry is integrated in these project teams and follows a project from start to finish, rather than being focused on a particular computational technique across many projects. Other pharma companies may structure their groups differently and that will affect how a computational chemist interacts with the projects. Being a much larger organization also leads to a unique environment compared to the start-up or small software space. In comparison to what Caitlin and Fio mentioned, there is an increased formality due to the size of the company and also more distance to organizational leadership. There are a lot of different groups that can be more focused on a particular problem or strategy, and this provides an opportunity to really develop some specialized expertise. The size of the organization and existence of specialized technology teams also means we have access to a very wide range of possible tools and software packages.”

### Starting out in Your Career

#### What Was the Most Surprising Thing When Starting in Industry?

##### Andrea (Large Pharma)

“I have been surprised by the amount of independence I have in decision making, as I have a lot of freedom to choose what tool to apply to a given problem or try new forms of analysis if I think they will be helpful. I was also surprised by the amount of different groups I ended up working with across different projects and initiatives. Additionally, especially in a large organization, it was surprising to realize that two people or groups that I worked closely with may have never interacted with each other.”

##### Fio (Start-up)

“I was surprised how I passively learned about other domains than my own by just being exposed to seminars and my colleagues talking about their research. Also, there are many acronyms, and I was bewildered by them initially and had to learn them. Many of them are company-specific.”

##### Caitlin (Software)

“I was surprised early on by how valued my opinion on a project was. I had a lot to learn, and I expected to not be able to contribute significantly until I had more experience. I have learned to value the different perspectives everyone brings to a project. When you are trying to make progress quickly, it’s important that everyone shares their observations, ideas, and concerns, because we will all think about problems in different ways.”

#### What Skill Set Did You Need for This Position?

##### Andrea (Large Pharma)

“An understanding of the fundamental principles in computational chemistry and intermolecular interactions is key. That helps you to be able to interpret results according to the scientific question at hand, even when using new software or techniques. My education was much more focused on quantum mechanics, and I needed the ability to pick up on new tools. A willingness to learn, combined with the ability to work independently, communicate clearly, and collaborate with others are just as important as scientific knowledge since a lot of the specifics are learned on the job.”

##### Caitlin (Software)

“Both of those soft skills and the fundamentals of computational chemistry translate into my setting as well. Additionally, advanced code development skills in the relevant language for the position were crucial. For instance, I develop in Python, but other positions require C++ as well.”

##### Fio (Start-up)

“Similar to Andrea, a good foundation in your specialty, which in my case is cheminformatics and drug discovery, is essential. At a start-up, it is likely you will be the only expert and will have to inform decisions with your expertise. In order to best lead such efforts, you need to be able to reach out to your network and other experts to get feedback. Finally, knowledge of open source packages is an advantage at a start-up as there may not be enough budget to purchase all the desired commercial software. This depends on which start-up and what their focus and needs are.”

#### What Are the Main Skills You Developed Early on?

##### Fio (Start-up)

“I found that the most important skills I developed starting out were not new technical skills but rather skills for communication and working in an industry environment. I was the only expert in my field within the company, so I needed to develop confidence to speak up and be able to quickly convey key analysis and work effectively with both my coworkers and external people from contract research organizations or other partners. I also had to develop flexibility and the ability to stretch into new areas. As an example, my chemistry background made me the go-to person for compound related questions at insitro initially, including regarding things like screening library storage conditions, despite the fact that my experience was not experimental, and I helped figure out that relevant information.”

##### Andrea (Large Pharma)

“I also found a lot of my growth at first was focused on confidence and communication, specifically communicating with people of more diverse scientific backgrounds. I learned how to process new types of information and work from less complete data as well. Simultaneously, I have expanded the number of software and computational chemistry tools I know how to use, generally picking them up as the situation may call for it. Learning a new tool involves both the technical learning of how to run it and how to communicate the key information in a meaningful way.”

##### Caitlin (Software)

“Like Fio and Andrea, confidence, trusting my instincts, and speaking up were also key things for me early on as well. In contrast, I also found learning new technical skills to be highly important. My previous work had been focused on classical force fields and small molecules in solution, so I had a lot to learn about CSP and QM calculations. However, I did find that my expertise in understanding molecular interactions translated to this new area. Generally, the ability to transfer skills to a new discipline and adapt to new techniques is really important in most industry positions.”

#### What Was Your Transition from Academia to Industry Like?

##### Fio (Start-up)

“My case may be a little special as I did my postdoc in industry, so I started my transition then. I had different collaborators throughout NIBR and had to learn to manage expectations and hone my ability to keep projects on track. This was useful as I transitioned into my positions at both start-ups. Another important aspect is being independent, addressing new problems while contacting potential vendors or partners when necessary. Each company I have been at had a formalized goal setting period for projects but also personal goals. The latter enables one to think about career development and build a plan with your manager, which I have found to be very different from my experience as a PhD student.”

##### Caitlin (Software)

“Somewhat similarly, I had interned with OpenEye during graduate school, which helped me understand what a career in a software company might look like. In addition, I had experience developing open source software, including feature planning, testing, and documentation as a part of the Open Force Field initiative. However, a major difference at OpenEye was accomplishing these tasks with shorter and stricter deadlines.”

##### Andrea (Large Pharma)

“While my position at Pfizer is my first industry job, I treated grad school like a job, already working consistent hours with clear work life boundaries, which made the transition into office life a lot easier. In addition to some of the changes discussed by Caitlin and Fio, I found that the biggest difference has been working in larger teams. This meant I needed to develop new systems for managing the inflow of data, communication, and tasks for different projects. While I am still fine-tuning these systems, I have started actually using Outlook color-coding, have improved my file naming systems and organization, and have found a combination of digital and handwritten notes that works for me to keep track of actions and outcomes from a meeting.”

#### How Did Your Company Help You Develop New Skills When Starting out?

##### Caitlin (Software)

“In starting my position at OpenEye, I had a big shift in the scientific area in which I was working. I was a good fit for the position because the group needed someone with a stronger chemistry background to help with improving the CSP models; however, I had a lot to learn to catch up. I attended conferences and webinars early on to help learn about the state of the art in CSP. This included learning how other computational groups approach the problem and the specifics of what problems our customers are trying to address.

Internally, we do a lot of learning by doing. For example, we have big group meetings to test our software where I learned about everything my group does by testing new features and reading documentation. I also picked up new skills by talking to my co-workers, in my group and outside of it, about what they were working on and how our customers used the software. When I started, I was given smaller scale projects to get used to the infrastructure and have taken on more responsibility with time.”

##### Andrea (Large Pharma)

“The training at Pfizer when I started out was pretty standardized and formal. I had a lot of specific training sessions including human resources trainings and software specific webinars. I also took a week-long internal course on medicinal chemistry for drug discovery right after starting, and later, a two day course on small molecule ADME, both run and taught by experienced people from within Pfizer. I have had a lot of opportunities to learn from more experienced computational chemists at Pfizer as I was initially paired up with another computational chemist on a project to learn the ropes and was partnered with a mentor in another group to discuss project problems and other work related topics. There are also opportunities to learn more about the biology and business sides of the organization through internal seminars.”

##### Fio (Start-up)

“Similar to Andrea, I also had several internal standardized human resources training sessions when I joined NIBR. Additionally, I took an online medicinal chemistry course to bring myself up to speed. My colleagues took a lot of time out of their schedule to teach me. In particular, at DiCE, I had to understand the DEL platform they had been building for the past 5 years. At both NIBR and DiCE, we went through personality evaluations early on and learned what types of personality our colleagues were in order to find out the most efficient ways to communicate with them and understand their concerns better. For instance, I learned how my main mentor appreciated having details about the research when presenting my work, while my second mentor needed rather brief on-point statements. I also learned a lot about myself, and it made me reflect on how I react to various work situations.”

### Going Forward

#### What Are the Career Progression Possibilities?

##### Caitlin (Software)

“The structure at OpenEye is quite flat; for example, I am in a group of about four people and my manager reports directly to our Chief Scientific Officer. This means career progression focuses on changes in your responsibilities, rather than focusing on moving up a ladder. These changes include expanding responsibilities while managing projects. For example, you may start in a role where you only make approved changes to a software package and move into a position where you manage a section of the software, making decisions about what features should be added to the next release or longer term prioritizing for expanding and optimizing that tool. Increased responsibilities can also translate to research, where more senior scientists are tasked with answering broader questions.”

##### Andrea (Large Pharma)

“Similar to what Caitlin described, the reporting structure within the computational chemistry group at Pfizer is pretty flat, and so, progression is more about responsibilities. You can be promoted in title while remaining focused on supporting drug discovery projects, but that comes with an expectation of increased involvement in decision making and project leadership as well as in cross-discipline initiatives. The responsibilities and expectations that come along with different titles are clearly laid out, and demonstrating that you are contributing at a higher level consistent with one of those descriptions can help you if you are working towards a promotion. Longer term, you can choose if you would rather focus on developing specific technical expertise or be involved in broader leadership, and some people even make changes that involve movement within the company to entirely new areas.”

##### Fio (Start-up)

“This is consistent with my experience as well. We also have internal ladders that describe roles and responsibilities for different career paths and that describe what skills you should demonstrate to obtain a promotion. There are tracks for scientists or moving into management, along with opportunities to switch departments, for example, moving to the data science department to be an engineer.”

#### How Does Your Company Support Your Career Development?

##### Andrea (Large Pharma)

“Career development is really an emphasis at Pfizer, including regular conversations with managers about what I want my career to look like. We are paired up with mentors in a variety of types of roles to learn more about the skills that are required. I look for opportunities to try new things, including learning new leadership and communications skills. We also have organizational support to go to workshops or conferences focused on these topics, and occasionally, speakers are brought in for internal career workshops. There are a lot of people with many experiences who are very willing to talk about the different aspects of their career, but it is up to you to seek out those conversations.”

##### Caitlin (Software)

“I also have regular conversations with my manager about the next steps of my career. However, one key difference is that at a smaller company there is not always going to be someone internally who has the skill that you want to develop. We are encouraged to pursue new scientific directions within our group’s focus areas and supported if there are conferences or training programs that we believe would help build skills for the next steps in our careers.”

##### Fio (Start-up)

“In smaller companies, people are more likely to take on new and different roles. I’ve felt that the company is very flexible, and several people have transitioned into different departments while I have been at insitro. My manager is open to new ideas or identified needs. For example, I suggested building a compound database and was given time to work on it. One needs to have the drive and be responsible in carrying each project forward.”

#### How Is Job Security?

##### Fio (Start-up)

“This is a question I get asked a lot, in particular regarding whether jobs at start-ups are less secure than jobs at more established and larger companies. It is a controversial question, but we would like to address it. One never really knows what’s going to happen. My general answer to this question is that, although the pharmaceutical industry is relatively robust to economic crisis, restructuring happens in all types of companies in the industry and at any economic standing of the company, so you may be laid off regardless of the size of the company. Large companies tend to lay off large sections at once, while start-ups may have a higher failure rate. The best prevention is to stay on top of the latest developments in your field and make sure your skills stay up-to-date. I have experienced a lay off, and I received a lot of support from the computational chemistry community and colleagues to find my next position.”

##### Caitlin (Software)

“In addition, the community of computational chemists, especially those working in drug discovery, is very small and tight knit. Maintaining and growing your network can help immensely in multiple areas of your career. Personally, I have leveraged my network by encouraging people I knew to apply to positions at OpenEye.”

##### Andrea (Large Pharma)

“I agree that there is no way to really know what the future holds and that maintaining your network and skill set is the best way to be able to weather any kind of change. That change may look different in different sectors. For example, while Pfizer as a company has been around for over a hundred years, in that time, there have been many changes in what diseases the company was focused on and in the geographic locations of the research facilities. These kinds of changes also require flexibility and the ability to transfer your skill set to new kinds of scientific problems.”

#### What Do You Wish You Had Known before?

##### Fio (Start-up)

“I wish I had had a better understanding of compensation packages, in particular stock options. Start-ups often come with an offer of stock options that vest according to a schedule. Also, it would have been good to spend more time at the beginning learning about the resources and benefits available to me; I am still learning about new benefits.”

##### Caitlin (Software)

“The company culture and your relationship with the coworkers on your team and outside it are important. Many people undervalue the importance of liking the people you work with as much as the job you do; I really appreciate the community at OpenEye.”

##### Andrea (Large Pharma)

“I’ve been surprised and impressed by people’s willingness to teach and answer questions; you definitely don’t need to figure everything out by yourself. My network has grown a lot since I began working, and I have definitely seen how growing your network can be incredibly important in navigating new situations and transitions.”

## Conclusion

We hope this Perspective helps you understand how industry positions vary on the basis of the size of the companies, the specific industries, or even the position. As disclosed throughout, this panel discussion reflects our personal perspectives and experiences specific to the companies where we have worked. The key similarities and differences are summarized in Table 1. Similarities are obvious in answers that are the same for all three columns, such as our interactions with many internal groups or the importance of knowing computational chemistry fundamentals to all positions. Differences can stem from the specific industry (e.g., responsibilities, where the software sector is quite different from either the start-up or large pharma) or be based on the size or structure of the company (e.g., career development, where the flat structure is common between a start-up pharma and a small software company, while large pharma is different).

**Table 1:** Summary of Key Similarities and Differences

	Andrea	Fio	Caitlin
sector	large pharma	start-up	software
company	Pfizer	insitro	OpenEye
# of employees	>70 000	∼150	∼100
age of company	>100 years	3 years	∼25 years
responsibilities	project support; validation of new tools	project support; computational infrastructure development; communication with vendors and collaborators	software coding, testing, and documentation; research new methods and techniques
groups/interactions	various internal teams	various internal teams; external vendors and collaborators	various internal teams; external customers and collaborators
skills needed	computational chemistry fundamentals; flexibility; communication; learning quickly	computational chemistry fundamentals; leadership; open source packages	computational chemistry fundamentals; coding (languages required may vary)
new skills	confidence; communication; new software	confidence; communication; flexibility; leadership	confidence; communication; new technical concepts
skill development	internal courses and webinars; formal and informal mentorship	external/online courses and webinars; informal internal knowledge sharing	conferences and webinars; informal internal knowledge sharing
career development	increase breadth of influence; responsibility within group	flat structure; increased responsibility	flat structure; increased responsibility

Hopefully, the descriptions of our experiences are useful to set you up to ask the questions that are the most important to you when looking for your next position. We want everyone to be able to find a role that fits their skill set, that they find fulfilling, and that aligns with their personal and professional goals. There are many factors involved in making that decision such as the size of the company, location, culture, or responsibilities, so understanding yourself and your values and staying committed to your own priorities are key. When you are looking for a job, keep in mind that everyone will have different perspectives about companies and types of industries. No one type of company is going to be the best fit for everyone, and we hope you can learn from our perspectives and experiences as you consider your own options.
